# The Reform of Prison Life

**Published:** 1920-08-14

**Authors:** 


### THE PUBLIC WELL-BEING.

### The Reform of Prison Life.

### WHAT IS CRIMINALITY?

The latest report of the Penal Reform League is a notable document which intimately concerns the moral and physical well-being of the nation. The British public knows very little about our criminal administration, and, with the exception of a small, inarticulate, and quite uninfluential section of the community, still less about what goes on inside the walls of a prison. But a few gleams of light on the subject are now beginning to attract public attention, because in recent years men of intellect and character whose opinions, however wrong-headed they might be, have brought them into conflict with the State have experienced the rigours of prison life and have the ability and the will to write about their experiences. We shall never master the problem of criminality until drastic reforms are carried out both in the administration of justice in the courts of law and in the conditions of prison life. With all the good will in the world, magistrates and judges are unable to act with proper discrimination under the present system, and once a man is made a convict his fate seems to be sealed for life.

Far-reaching improvements may be effected when full advantage is taken of the body of knowledge made available by modern psychological research. As Dr. James Glover points out, to punish a mentally defective person is just about as rational and sensible as to flog an Australian aborigine for failing to comprehend Einstein's theory of light, and it is common knowledge, in the medical profession at any rate, that the ranks of so-called criminals contain a very large proportion of persons who are really mentally defective or who are in some cases actually insane. The study of the subconscious processes, both of abnormal and so-called normal individuals, has revealed the fact that practically none of us is acquainted with the inner motives for action, and that the explanations we offer and which we genuinely believe for doing many things are, on investigation, shown to be largely determined by motives entirely sub-conscious. "In face of the fact that this occurs in spite of the best mental endowment and the advantages of a sympathetic upbringing, how can we be other than patient and charitable," asks Dr. Glover, "to those whose powers of understanding and of controlling the tremendous pent-up energies within them are weakened by inherent instability and perverted by conditions of life which in many cases expressly foster and encourage a return to the discarded code of the jungle?" Surely here is opened up a wide field for scientific treatment.

As for the conditions of life in prison, even the warders, who are not usually regarded as tender-hearted beings, give testimony that it has a brutalising effect, and is neither deterrent nor preventive. One prison officer said to a member of the League: "I would rather have my boy dead than have him in this prison, because in the case of nine out of ten of the boys here their after-record is that they are in and out, in and out. They become experts in criminality by the treatment from which they suffer here, and by the experience which they gain here."

#### Peril of Women's Prisons.

A number of ladies who have had experience of women's prisons told Mr. Fenner Brockway that "so far as the young girls are concerned women's prisons are little more than educational establishments for prostitutes." It is still too generally assumed that a convict is *ipso facto* a bad man, representing in himself the concentrated essence of original sin. Yet careful examination tends to prove that most criminals are the victims of circumstances, or of physical or social conditions—men who, like the hero of Mr. Galsworthy's "Justice," having fallen, through stress of poverty or misfortune, into one dishonest act, had immediately been caught into the penal system which, once having trapped a man, "rarely fails to turn him into an habitual criminal."

